# Ultrasound and Shear Wave Elastography of Lower-Limb Muscles and Aponeurotic Structures in Human Cadavers—A Scoping Review

**DOI:** 10.3390/diagnostics16101571

**Published:** 2026-05-21

**Authors:** Filippo Tilli, Giorgio Tamborrini, Felix Margenfeld

**Affiliations:** 1 Medicina e Scienze della Salute Vincenzo Tiberio, University of Molise, 86100 Campobasso, Italy; 2 Institute of Rheumatology, University of Basel, 4056 Basel, Switzerland; sonoanatomy@pm.me; 3 Department of Biomedicine, University of Basel, 4056 Basel, Switzerland; felix.margenfeld@unibas.ch

**Keywords:** scoping review, human cadaver, ultrasound, shear-wave elastography, lower-limb muscles, aponeurosis, sports injuries

## Abstract

**Background**: Human cadaveric models provide a controlled experimental setting to investigate the anatomical basis and mechanical behaviour underlying musculoskeletal ultrasound findings. In recent years, both B-mode ultrasound and shear wave elastography have been applied in cadaveric studies to explore muscle architecture, aponeurotic structures, and passive mechanical properties under standardized conditions. **Objective**: The aim of this scoping review was to map and synthesize cadaveric studies using ultrasound and shear wave elastography to investigate lower-limb muscles and their aponeurotic structures, with emphasis on methodological applications, anatomical insights, and limitations relevant to clinical interpretation. **Material and Methods**: A scoping review was conducted according to PRISMA-ScR principles. Studies were included if ultrasound imaging (B-mode and/or shear wave elastography) was applied directly to human cadaveric lower-limb muscles or aponeurotic structures. Data were extracted and synthesized descriptively by anatomical region and ultrasound technique. **Results**: A total of 11 studies met the inclusion criteria and were included in the final qualitative synthesis, all of which applied ultrasound imaging, with or without shear wave elastography, directly to human cadaveric muscle tissue Among these, seven studies specifically investigated lower-limb skeletal muscles and their aponeurotic structures using ultrasound-based techniques to describe muscle architecture, internal connective tissue anatomy, or passive mechanical behaviour. These studies focused on the quadriceps femoris, hamstrings, adductor longus, and the gastrocnemius–soleus complex. The remaining four studies were considered relevant and therefore included in the scoping review because, although they did not focus on a specific lower-limb muscle group, they addressed key methodological factors influencing ultrasound and elastography-derived measurements in cadaveric muscle tissue. These investigations examined the effects of tissue layering, specimen-related characteristics, and measurement conditions, thereby providing essential methodological context for the interpretation of ultrasound-based outcomes across different anatomical regions. **Conclusions**: Cadaveric ultrasound studies provide essential anatomical context for interpreting musculoskeletal ultrasound, while cadaveric shear wave elastography supports controlled exploration of passive muscle mechanics. At the same time, these studies highlight important methodological sensitivities that should be acknowledged before translating elastography findings to clinical decision-making.

## 1. Introduction

Lower-limb muscle injuries represent a major challenge in sports medicine and are particularly prevalent in football, where they are associated with high recurrence rates and substantial time loss [1]. Despite advances in imaging and rehabilitation strategies, recurrent injuries remain common, indicating that relevant structural and mechanical aspects of muscle damage are still incompletely understood.

From a pathomechanical perspective, sports-related muscle injuries occur predominantly at regions of force transmission rather than within the muscle belly itself. The myotendinous junction represents a transitional zone where contractile muscle fibers integrate with connective tissue to transfer force to tendons and aponeuroses. In this context, the organization of intramuscular connective tissue and the extracellular matrix plays a central role in modulating strain distribution and mechanical load, particularly during high demand activities such as sprinting and kicking [2,3].

In several lower-limb muscles, force transmission is organized around intramuscular or central tendinous structures that act as internal load-bearing elements. While this hierarchical architecture allows efficient force transfer, it also creates mechanical heterogeneity within the muscle, potentially contributing to injury-prone interfaces and recurrence. Understanding these internal structural arrangements is therefore essential for interpreting injury patterns beyond isolated fiber disruption [4,5,6].

Cadaveric studies offer a specific advantage for investigating these mechanisms. Unlike in vivo investigations, cadaver models allow direct correlation between imaging findings and true anatomy under standardized experimental conditions, providing anatomical and methodological reference frameworks that cannot be fully obtained in living subjects [7].

Musculoskeletal ultrasound has become a cornerstone in the diagnostic assessment of lower-limb muscle injuries due to its real-time, dynamic capabilities, high spatial resolution for superficial and deep structures, and wide availability. However, conventional B-mode ultrasound primarily provides morphological information and offers limited insight into tissue mechanical behaviour.

In this context, elastography represents an important adjunct to B-mode imaging. By assessing tissue stiffness based on rheological principles, shear wave elastography provides complementary information on the mechanical properties of muscle and connective tissue, extending ultrasound assessment beyond morphology alone [8].

However, elastography findings obtained in cadaveric tissue are strongly influenced by specimen preservation methods, tissue hydration, passive prestress conditions, anisotropy, and wave propagation behaviour. Formalin fixation, freeze–thaw cycles, and Thiel embalming may differently alter tissue viscoelastic properties and Young’s modulus measurements [9,10,11,12]. Consequently, cadaveric SWE findings should primarily be interpreted as methodological and biomechanical reference data rather than direct surrogates of in vivo muscle stiffness.

The aim of this scoping review is therefore to map and synthesize cadaveric studies using ultrasound and shear wave elastography in lower-limb muscles, to clarify their anatomical relevance, methodological applications, and translational limits for musculoskeletal imaging.

## 2. Methods

### 2.1. Study Design

This scoping review was based on a search on the use of Ultrasound on human cadaveric muscles and was conducted in accordance with the Preferred Reporting Items for Systematic Reviews and Meta-Analyses extension for Scoping Reviews (PRISMA-ScR) guidelines [13]. To fulfill the quality criteria for good scientific practice, a protocol for the review was registered on OSF Registries (osf.io/mx468) on 28 November 2022, accessed on 18 May 2026. The full search strategy, study selection details and Tables S1 and S2 are provided as Supplementary Material and Appendix A Table A1.

### 2.2. Eligibility Criteria

Studies were included if ultrasound imaging (B-mode and/or shear wave elastography) was applied directly to human cadaveric lower-limb muscles or aponeurotic structures.

### 2.3. Search Strategy

The literature search was conducted in PubMed using combinations of terms related to ultrasonography, cadaveric specimens, and musculoskeletal anatomy. The complete search strategy is reported in Appendix A Table A1. Searches were updated until February 2025. Studies were eligible when B-mode ultrasound and/or shear wave elastography (SWE) were directly applied to human cadaveric lower-limb muscles or aponeurotic structures. Animal studies, transplantation-related studies, and studies unrelated to lower-limb musculoskeletal anatomy were excluded. The review process followed PRISMA-ScR recommendations to improve methodological transparency and reproducibility.

### 2.4. Study Selection

All records were managed using EndNote X9 (Clarivate, Philadelphia, PA, USA), with duplicates removed automatically following Bramer et al. [14] and manually thereafter. A broad search strategy was applied. Titles and abstracts were screened in EndNote against predefined inclusion criteria for ultrasound studies on human cadavers by one reviewer, with consultation of a second reviewer when needed. Full-text screening was conducted independently by two reviewers. Included studies were organized by anatomical focus. A classification system of 12 categories and 239 sub-categories was developed (Table S2, Supplementary Material) Studies classified under “muscles” were independently reviewed in full text by two reviewers to identify ultrasound investigations of the muscles. Discrepancies were resolved by a third reviewer.

### 2.5. Data Extraction and Synthesis

For each included study, information was collected on the authors, publication year, title, investigator, equipment, procedure description, measurement, and outcome. Data extraction was carried out independently by two reviewers, after which the results were combined into a single table highlighting the main findings. In addition, the outcomes of all included studies were qualitatively synthesized through a narrative review. Any discrepancies between the reviewers were resolved through discussion and consensus.

## 3. Results

### 3.1. Search and Selection of Included Studies

**Figure 1:**
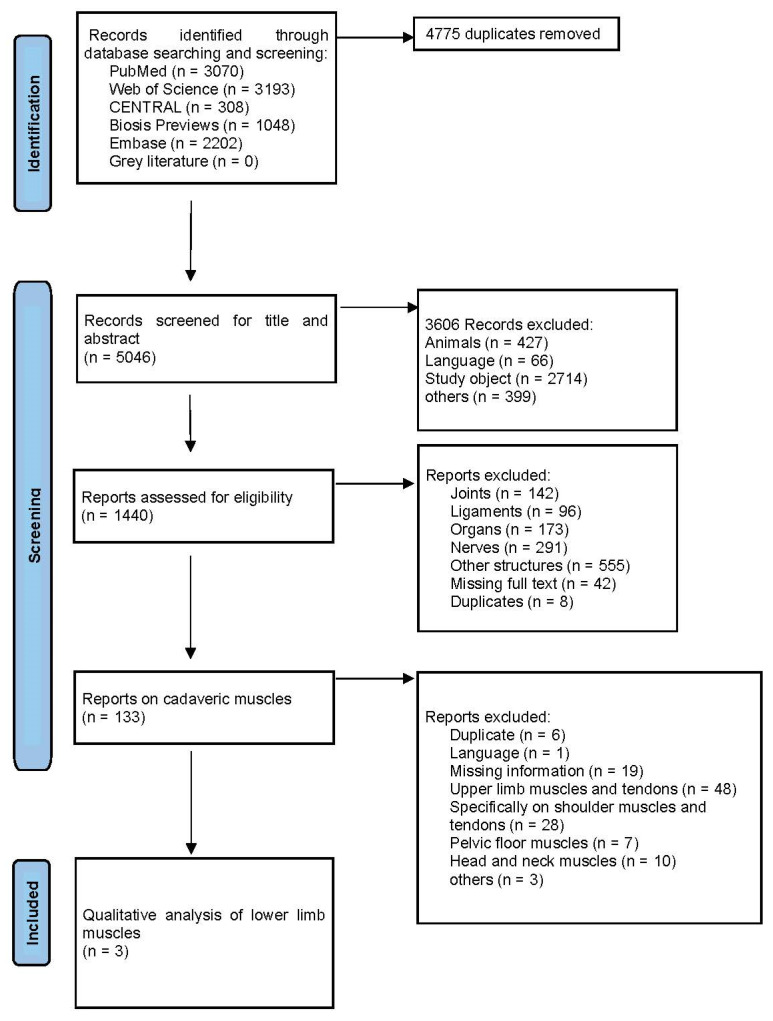
PRISMA Flowchart.

The literature search identified 9821 records, of which 4775 were duplicates and were therefore removed. Following duplicate removal, records underwent title and abstract screening, after which full-text assessment was performed according to the predefined eligibility criteria. Among these, 133 publications were assigned to the “muscles” category, and 11 studies ultimately met the inclusion criteria. The study selection process is summarized in the PRISMA flowchart (Figure 1).

### 3.2. Study Characteristics of Included Studies

The 11 included studies were published between 1996 and 2022, were written in English, and were published as full journal articles [15]. All studies employed cadaveric specimens either to validate ultrasound findings against anatomical reference standards or to allow controlled experimental conditions not achievable in vivo [16].

B-mode ultrasound was primarily used to depict intramuscular connective tissue structures and to assess muscle architecture under standardized acquisition settings [17]. Shear wave elastography (SWE) was applied in a subset of studies to explore passive mechanical behaviour of muscle tissue during controlled loading or under defined tissue-layer conditions [18]. Study designs included cadaveric ultrasound combined with anatomical correlation through dissection or section-based comparison, ex vivo passive loading experiments with ultrasound-based measurements, and hybrid approaches integrating cadaveric validation with in vivo or clinical imaging components [16]. Seven studies focused on lower-limb muscle groups commonly examined in the context of sports-related muscle disorders, including the quadriceps, hamstrings, adductors, and calf muscles [19]. The remaining studies addressed methodological determinants relevant to musculoskeletal ultrasound and elastography measurements across muscle regions [20].

### 3.3. Rectus Femoris and Quadriceps Region

A cadaveric anatomical study clarified the internal architecture of the rectus femoris and validated the sonographic appearance of the central aponeurosis through direct anatomical correlation [16]. A second investigation established a dedicated ultrasound approach for the indirect tendon and confirmed its identification by cadaveric needle-guided validation with histological and anatomical reference [21]. One cadaveric shear wave elastography experiment examined the rectus femoris under incremental passive loading, acquiring repeated measurements at predefined sites along the muscle length [18]. In addition, a cadaveric B-mode study evaluated the influence of overlying tissue layers on quantitative grayscale outcomes using stepwise tissue removal models at the anterior thigh [20].

### 3.4. Hamstrings

A cadaver-validated ultrasound study described the segmental morphology of the long head of the biceps femoris using standardized multi-level acquisitions along the muscle length [19]. A second cadaveric validation study compared ultrasound-derived architectural parameters with dissection-based measurements obtained under fixed joint positioning [17].

### 3.5. Calf (Gastrocnemius–Soleus Complex)

A combined cadaveric and clinical investigation defined the relevant calf anatomy on cadavers and used this anatomical reference to interpret ultrasound patterns observed in a large clinical cohort with suspected “tennis leg” [22]. A cadaveric shear wave elastography study demonstrated that stiffness measurements in the medial gastrocnemius are strongly influenced by superficial tissue layers, with a marked reduction following skin removal [23]. In addition, a cadaver-based procedural study mapped key sono-anatomical landmarks for proximal gastrocnemius recession and verified procedural safety through post-procedural anatomical inspection, followed by clinical application [24].

### 3.6. Adductors

A cadaveric study investigated the adductor longus under controlled passive loading and reported a consistent increase in shear elastic modulus across predefined regions [25].

### 3.7. Cadaver Type

Soft-embalmed cadavers, predominantly preserved using the Thiel method, were mainly employed in studies investigating passive mechanical behaviour through shear wave elastography because this preservation technique better preserves tissue flexibility and joint mobility compared with formalin fixation [18]. Nevertheless, previous biomechanical investigations have demonstrated that Thiel preservation still alters tissue viscoelastic properties and Young’s modulus measurements, with stiffness progressively changing over time after embalming [9,10,11,12]. Fresh-frozen cadavers were preferentially used in studies requiring high-fidelity anatomical correlation between ultrasound findings and native tissue morphology [22]. Formalin-fixed cadavers were primarily used for structural and anatomical validation studies, although formalin fixation is known to substantially increase tissue stiffness and alter acoustic properties relevant to elastography measurements [21]. These preservation-related factors represent important methodological limitations when interpreting cadaveric SWE findings and comparing results across studies.

### 3.8. B-Mode Ultrasound Visualization and Anatomical Correlation

Across anatomical regions, B-mode ultrasound allowed visualization of aponeuroses, intramuscular tendons, and myotendinous interfaces when probe orientation followed true anatomical planes [16]. Cadaveric reference standards supported the anatomical credibility of architectural measurements obtained by ultrasound in the hamstrings [17]. In the calf, ultrasound reliably differentiated the medial gastrocnemius-, soleus-, and plantaris-related structures when interpreted against cadaveric anatomy [22].

### 3.9. Shear Wave Elastography Findings

SWE experiments consistently used controlled cadaveric conditions to relate passive loading or tissue-layer conditions to elastography-derived stiffness readouts [18]. In the adductor longus, passive loading produced a clear, progressive increase in shear elastic modulus across the sampled regions [25]. In the rectus femoris, elastography acquired along the longitudinal fiber direction tracked passive loading more consistently than measurements obtained outside the fiber aligned orientation [18]. In the medial gastrocnemius, removing the skin produced a substantial reduction in measured shear modulus, highlighting the effect of superficial layers on muscle stiffness readouts [23].

## 4. Discussion

This scoping review demonstrates that cadaveric ultrasound studies provide a robust anatomical and methodological framework for interpreting musculoskeletal ultrasound findings in lower-limb muscles. These investigations clarify how internal architecture, connective tissue organization, and acquisition conditions shape both B-mode and elastography-derived signals, thereby supporting more anatomically grounded image interpretation.

A central finding of the reviewed literature concerns the role of intramuscular connective tissue structures in injury-prone regions. Cadaveric validation shows that B-mode ultrasound can reliably depict aponeurotic and intramuscular tendon components when imaging planes are aligned with true anatomy [16]. This supports an anatomy-driven interpretation of muscle injuries, particularly in cases characterized by persistent symptoms or recurrent episodes despite limited fiber disruption on imaging. From a clinical perspective, focused assessment of myo-aponeurotic interfaces may therefore improve diagnostic accuracy and follow-up evaluation in high-risk muscles.

Across lower-limb muscles, cadaveric studies consistently demonstrate strong agreement between ultrasound-derived architectural parameters and direct anatomical measurements under controlled conditions [17]. These findings support the use of ultrasound for structural assessment of myotendinous regions, while emphasizing that accurate interpretation depends on detailed knowledge of internal muscle organization [19]. In the calf, anatomical correlation has clarified that acute injuries commonly referred to as “tennis leg” predominantly involve the medial gastrocnemius myotendinous junction, often accompanied by inter-aponeurotic fluid, rather than isolated rupture of the plantaris tendon [22]. This distinction has direct implications for diagnostic focus and clinical management.

Shear wave elastography emerges from the cadaveric literature as a valuable adjunct to B-mode imaging rather than a standalone technique. Under controlled experimental conditions, SWE measurements demonstrate sensitivity to passive loading and prestress states. However, this relationship should not be interpreted as a direct measurement of intrinsic tissue material properties alone. In skeletal muscle, passive tension itself alters shear wave propagation behaviour through acoustoelastic effects [26,27], meaning that increases in measured shear modulus may partially reflect load-dependent wave propagation phenomena rather than isolated changes in intrinsic muscle stiffness. Additional complexity arises from the strongly anisotropic and waveguide-like behaviour of skeletal muscle tissue [11,12]. When muscle dimensions become comparable to shear wavelength, reflected and guided waves may substantially influence elastography measurements. Intramuscular tendons, pennate architecture, and aponeurotic interfaces further contribute to heterogeneous propagation patterns and regional variability in stiffness estimates.

These observations reinforce the importance of interpreting SWE as a complementary and method-sensitive technique rather than a direct standalone biomarker of intrinsic muscle stiffness [12,26]. Accordingly, elastography measurements should be interpreted within the biomechanical and anatomical context in which shear waves propagate, rather than as isolated material constants. This distinction is particularly important in cadaveric investigations, where preservation-related alterations and controlled loading conditions may substantially influence shear wave behaviour independently of native physiological muscle function.

At the same time, cadaveric and ex vivo studies highlight important technical and anatomical limitations that constrain the reliability, comparability, and clinical interpretability of shear wave elastography [9]. Differences in hardware implementation, push pulse generation, tracking algorithms, and vendor specific presets lead to systematic variability in shear wave speed and stiffness values across platforms, limiting direct comparison between systems even under controlled experimental conditions [9,10]. Acquisition-related factors such as probe pressure, region of interest size, insonation angle, and measurement depth further influence stiffness estimates, particularly in superficial or mechanically heterogeneous tissues [9,28]. Reduced penetration and increased attenuation in deeper muscles additionally compromise signal quality and reproducibility, especially when high-frequency linear transducers are used for musculoskeletal imaging [10,29].

Muscle specific structural properties introduce further complexity. Skeletal muscle, tendons, and aponeuroses are strongly anisotropic, and shear wave propagation depends critically on fiber orientation relative to the transducer [10,26,29]. Small deviations in probe alignment can therefore lead to marked changes in measured stiffness, reducing inter-session and inter-operator reproducibility [10,30]. Intramuscular tendons, pennate architecture, and aponeurotic interfaces generate heterogeneous propagation pathways, producing spatially variable elastograms that reflect architectural complexity rather than intrinsic tissue stiffness alone [9,30].

Physiological and protocol-related factors further limit clinical translation. Importantly, SWE values obtained from cadaveric specimens should not be directly extrapolated to living subjects or interpreted as normative clinical values. Cadaveric elastography primarily provides controlled biomechanical and methodological reference data that support anatomical interpretation and experimental understanding of passive tissue behaviour. Shear wave elastography values increase with passive muscle stretch and even minimal muscle activation [12,27], independent of structural pathology. Minor variations in joint position, muscle length, or patient guarding can therefore overshadow disease-related differences, reducing diagnostic effect size at the individual level [28,29]. Although more rigorous positioning protocols can mitigate variability, they are time-consuming and difficult to implement consistently in routine clinical practice.

Taken together, these findings indicate that shear wave elastography should be interpreted as an anatomy dependent, method sensitive adjunct rather than a quantitative standalone biomarker. Within cadaveric research, elastography plays a valuable role in defining methodological boundaries and exploring passive mechanical behaviour under standardized conditions. In clinical practice, its greatest utility lies in supporting anatomically informed interpretation alongside B-mode ultrasound, while avoiding overinterpretation of stiffness values as direct surrogates of muscle function or injury severity [31,32].

Overall, the cadaveric literature indicates that ultrasound and elastography studies primarily serve as anatomy validating and method defining evidence. Their principal contribution lies in refining image interpretation, improving understanding of myo-aponeurotic injuries and preventing inappropriate extrapolation of passive ex vivo mechanical metrics to in vivo muscle function.

## 5. Conclusions

Cadaveric ultrasound studies provide important anatomical and methodological reference frameworks for interpreting musculoskeletal imaging findings in lower-limb muscles. B-mode ultrasound reliably depicts intramuscular connective tissue and myo-aponeurotic structures when interpreted with detailed anatomical knowledge. Shear wave elastography provides complementary information regarding passive mechanical behaviour under controlled experimental conditions; however, SWE findings are highly dependent on tissue preservation, loading conditions, anisotropy, and acquisition methodology. Therefore, cadaveric elastography findings should be interpreted cautiously and should not be directly extrapolated as normative indicators of in vivo muscle stiffness or function. The principal value of cadaveric ultrasound and elastography studies lies in improving anatomical interpretation and methodological understanding in musculoskeletal imaging.

## Data Availability

The original contributions presented in this study are included in the article. Further inquiries can be directed to the corresponding author.

## Supplementary Materials

The following supporting information can be downloaded at: https://www.mdpi.com/article/10.3390/diagnostics16101571/s1, Table S1: PubMed Search strategy; Table S2: List of anatomical structures used for the database. The number of sub-categories for each main category is provided in brackets; Supplementary Materials: Preferred Reporting Items for Systematic reviews and Meta-Analyses extension for Scoping Reviews (PRISMA-ScR) checklist.

## Appendix A

**Table A1:** Summary of the seven reviewed studies.

Author (Year)	Title	Sample Size	Investigator	Equipment	Procedure Description	Measurement	Outcome
Takuya Kato et al. (2022) [25]	Adductor longus: An anatomical study to better understand groin pain	9 AL muscles were harvested from Thiel soft-embalmed cadavers (mean age at death: 86 years; range: 73–98 years).	Research team from Sapporo Medical University with expertise in cadaveric biomechanics and shear wave elastography	Shear wave elastography with a linear ultrasound transducer (4–15 MHz) (Aixplorer Ver. 12 and SL15-4; Supersonic Imagine, Aix-en Provence, France) Ultrasound echo pad with a thickness of 30 mm (Echo PAD; Yasojima Proceed Co., Ltd., Kobe, Japan) Custom-built loading device with clamps, pulley, and cable to apply passive loads from 0 to 600 g in 60 g increments	The adductor longus (AL) muscles were meticulously dissected from Thiel-embalmed cadavers, preserving both proximal and distal tendons to maintain physiological fiber orientation and native muscle architecture. Each muscle specimen was mounted on a custom-designed loading apparatus that applied standardized passive tensile loads via a pulley–weight system. The muscle belly was positioned on an echo-absorptive pad to ensure consistent acoustic coupling and to minimize ultrasound reflection artifacts. Shear-wave elastography (SWE) measurements were performed with the ultrasound transducer aligned longitudinally with the muscle fibers, ensuring that shear-wave propagation occurred along the principal mechanical axis of the muscle. Using B-mode imaging, measurements were obtained at three anatomically defined regions along the muscle length: proximal, middle, and distal thirds. Passive loads were applied incrementally from 0 to 600 g in 60 g steps. Each load was maintained for less than 10 s to minimize viscoelastic creep. All measurements were repeated twice to assess test–retest reliability. Following mechanical testing, architectural parameters including muscle length, mass, and anatomical cross-sectional area were recorded for biomechanical correlation analyses.	Muscle shear modulus (kPa) was quantified at each load level using shear-wave elastography at the proximal, middle, and distal regions of the adductor longus. Force–stiffness relationships were derived by plotting shear modulus against applied passive load, allowing calculation of regional stiffness slopes (kPa/g). Test–retest reliability was assessed using intraclass correlation coefficients (ICC).	Across all regions of the adductor longus, shear modulus increased in a highly linear manner with increasing passive load, with coefficients of determination of 0.989 (proximal), 0.986 (middle), and 0.982 (distal). At slack length (0 g load), mean shear modulus values were highest proximally (11.83 ± 3.80 kPa), followed by the middle (10.00 ± 1.95 kPa) and distal regions (7.90 ± 2.00 kPa). The rate of stiffness increase per gram of applied load showed clear regional variation, being greatest proximally (0.056 ± 0.027 kPa/g), intermediate in the mid-region (0.044 ± 0.010 kPa/g), and lowest distally (0.038 ± 0.012 kPa/g). Test–retest reliability was exceptionally high (ICC > 0.99 at all regions). Correlation analyses revealed that baseline shear modulus was independent of muscle architectural parameters, whereas stiffness–load slopes were moderately to strongly associated with the reciprocal of muscle mass and anatomical cross-sectional area, indicating that smaller muscles exhibited proportionally greater stiffness increases under passive tension. Overall, the study demonstrated that the adductor longus exhibits a predictable and regionally consistent linear stiffness–force behaviour under passive loading. These findings validate shear-wave elastography as a reliable surrogate for estimating passive muscle force and provide important biomechanical insights into regional strain susceptibility and the mechanistic basis of adductor-related groin pain.
Yasuhide Yoshitake et al. 2015 [23]	The skin acts to maintain muscle shear modulus	6 lower limbs from 4 Thiel soft-embalmed human cadavers (3 ♂ male, 1 ♀ female; mean age at death: 89.3 ± 7.5 years)	Research team from the National Institute of Fitness and Sports in Kanoya and Sapporo Medical University, with expertise in musculoskeletal biomechanics, anatomy, and shear wave elastography	Ultrasound shear-wave elastography system (AixPlorer Ver. 6.3; Supersonic Imagine, Aix-en-Provence, France) Linear-array ultrasound transducer (4–15 MHz, SL15-4, 50 mm width) Built-in Q-Box™ software v. 6-3 for shear modulus quantification Goniometer for ankle joint angle control Surgical instruments for controlled removal of skin and epimysium	The medial gastrocnemius (MG) muscle was examined in Thiel-embalmed cadavers laid in the prone position on a specially designed bed, with the knees positioned at approximately 170° of extension [180° indicating full extension]. Shear-wave elastography (SWE) measurements were performed at rest with the ultrasound probe aligned longitudinally with the muscle fascicles to ensure accurate propagation of shear waves along the muscle fiber direction. Care was taken to avoid probe-induced compression. Measurements were obtained under three sequential experimental conditions: CONT: intact cadaver with skin and epimysium preserved; SKIN: complete removal of skin and subcutaneous tissue while preserving the epimysium; ALL: subsequent removal of the epimysium, leaving the muscle exposed. For each condition, three SWE images were acquired at 3 s intervals from the same anatomical location on the muscle belly, verified using B-mode imaging. In a second experiment, the effect of probe–fascicle angle on shear modulus was evaluated by positioning the probe either parallel to the fascicles (WO-angle) or obliquely (W-angle) at ankle joint angles of 10°, 20°, and 30° of plantarflexion.	Muscle shear modulus (kPa) was quantified as the spatial average within a circular region of interest (ROI) using SWE. Additionally, the angle between the probe’s transverse imaging axis and the muscle fascicle direction was measured from B-mode images using ImageJ software v1.53 (National Institutes of Health, Bethesda, MD, USA).	Removal of the skin resulted in a significant 50% reduction in medial gastrocnemius shear modulus compared with the intact condition (*p* < 0.01). No further significant change in shear modulus was observed after removal of the epimysium, indicating that the epimysium contributed minimally to maintaining resting muscle stiffness. Although removal of the skin also reduced pennation angle, controlled experiments demonstrated that changes in probe–fascicle angle accounted for only minor variations in shear modulus (~0.58 kPa per degree), insufficient to explain the large stiffness decrease. The study conclusively showed that skin is the primary tissue responsible for maintaining passive muscle shear modulus, acting as a mechanical constraint that preserves muscle stiffness and architecture. These findings provide important biomechanical insight into muscle–skin interactions and support the clinical relevance of SWE for assessing muscle mechanical properties in aging, injury, surgery, and rehabilitation contexts.
Manuel Villanueva et al. 2018 [24]	Proximal ultrasound-guided gastrocnemius recession: A new ultra-minimally invasive surgical technique	Cadaver study: 16 human cadavers (mean age at death: 57 ± 8 years; 11 males, 5 females) Clinical study: 12 patients (23 lower limbs; 6 males, 6 females; mean age: 42 years, range 31–55 years)	Multidisciplinary research team from the Avanfi Institute and Unit for Ultrasound-Guided Surgery (Madrid, Spain), including orthopedic surgeons and podiatrists with extensive expertise in ultrasound-guided minimally invasive procedures	High-resolution ultrasound systems for musculoskeletal and interventional imaging: Alpinion ECube15 ultrasound device (Alpinion Medical Systems, Gyeonggi-do, Republic of Korea) Linear-array transducer (10–17 MHz) Needle Vision Plus™ ultrasound-guidance software Ultrasound-guided surgical instruments: Hook knife V-shaped straight curettes (small and medium) Long needle guides Blunt dissectors	The study was conducted in two phases. In the cadaveric phase, proximal medial gastrocnemius recession was performed under continuous ultrasound guidance to evaluate the feasibility, accuracy, and safety of the technique. The procedure involved selective release of the proximal medial gastrocnemius aponeurosis through a single 1–2 mm skin incision, performed 2–3 cm distal to the popliteal crease. Careful ultrasound identification of adjacent neurovascular structures (sural nerve, saphenous vein, hamstring tendons) was used to prevent iatrogenic injury. In the clinical phase, ultrasound-guided proximal medial gastrocnemius recession was performed in patients with gastrocnemius contracture (ankle dorsiflexion <10° with knee extended), either as a standalone procedure or in combination with other ultrasound-guided interventions. All procedures were performed under local anesthesia with sedation, without tourniquet use or limb ischemia. The surgical approach consisted of transverse ultrasound probe placement, percutaneous creation of a working space beneath the superficial fascia, and controlled medial-to-lateral release of the gastrocnemius aponeurosis using a hook knife under real-time ultrasound visualization. Immediate post-release ankle dorsiflexion was assessed intraoperatively. No sutures were required, and early mobilization was encouraged.	Primary outcomes included: Ankle dorsiflexion range of motion (degrees) measured pre- and post-procedure Pain intensity assessed using the Visual Analog Scale (VAS) Functional outcome assessed using the American Orthopedic Foot and Ankle Society (AOFAS) Ankle-Hindfoot Score Secondary outcomes included complication rates, neurovascular integrity, calf muscle strength, and recovery timeline.	In the cadaver study, effective release of the proximal medial gastrocnemius tendon was achieved in all specimens, with no damage to the saphenous vein, sural nerve, hamstring tendons, or surrounding structures, confirming procedural safety. In the clinical cohort, ankle dorsiflexion increased by a mean of 12° (range 6–18°, *p* = 0.05) in all treated limbs and was maintained throughout follow-up. Pain levels improved significantly, with mean VAS scores decreasing from 7 (range 5–9) preoperatively to 1 (range 0–2) at 12 months (*p* = 0.01). Functional outcomes improved markedly, with AOFAS Ankle-Hindfoot Scores increasing from a mean of 25 (range 20–40) preoperatively to 85 at 6 months and 90 at 12 months (*p* = 0.01). No major complications were observed. Minor complications included transient superficial hematomas and one case of temporary sensory disturbance, which resolved over time. No calf weakness or overlengthening was reported. Overall, the study demonstrated that ultrasound-guided ultraminimally invasive proximal medial gastrocnemius recession is a safe and effective alternative to open surgical techniques. The approach offers significant advantages, including minimal incision size, reduced pain, avoidance of limb ischemia and deep anesthesia, rapid recovery, and high patient satisfaction, supporting its potential role in the modern surgical management of gastrocnemius contracture and associated lower-limb pathologies.
Marco V. Narici et al. 1996 [15]	In vivo human gastrocnemius architecture with changing joint angle at rest and during graded isometric contraction	In vivo study: 6 healthy adult males (age: 38.0 ± 8 years; height: 1.76 ± 0.05 m; body mass: 67.8 ± 6.5 kg) Cadaver validation: 1 human cadaver lower limb (62-year-old male)	Multidisciplinary research team from the Consiglio Nazionale delle Ricerche (Italy) and the University of Genova, with expertise in muscle physiology, biomechanics, radiology, and ultrasonography	Real-time B-mode ultrasonography system (Acuson 128XP; Acuson Inc., Mountain View, CA, USA) Linear ultrasound probe (7.5 MHz; 4 cm length) Magnetic resonance imaging (MRI) system (1.5 T; Picker International) for muscle volume and length assessment Electronic goniometer for ankle joint angle measurement Force transducer system for plantar flexion force measurement	The gastrocnemius medialis (GM) muscle architecture was examined in vivo using ultrasonography at rest and during graded voluntary isometric plantar flexion contractions up to maximum voluntary contraction (MVC). Participants were seated with the lower limbs fully extended and the foot secured to a footplate, maintaining a standardized ankle joint angle (≈110° during contraction trials). Ultrasound images were acquired along the median longitudinal axis of the GM, defined from the distal muscle belly to the proximal tendon, ensuring alignment with muscle fascicles. Measurements were obtained at three anatomically defined regions along the muscle length: proximal (P), central (C), and distal (D). For each region, pennation angle, fascicle length, and distance between superficial and deep aponeuroses were measured at rest and during isometric contractions of increasing intensity. To examine the effect of muscle length at rest, ultrasound measurements were repeated while systematically varying ankle joint angle from 90° to 150° in 5° increments. For methodological validation, the same architectural parameters were measured by ultrasound and then directly by anatomical dissection in a cadaver leg positioned identically to the in vivo measurements.	Primary architectural parameters: Pennation angle (degrees) Muscle fascicle length (mm) Distance between aponeuroses (mm) Secondary derived variable: Physiological cross-sectional area (PCSA), calculated from muscle volume, fascicle length, and pennation angle Force–architecture relationships were analyzed across contraction intensities and joint angles.	At rest, increasing ankle joint angle from 90° to 150° resulted in a significant increase in pennation angle (from ~15.8° to ~27.7°) and a concomitant decrease in fascicle length (from ~57.0 to ~34.0 mm), leading to a 51% increase in PCSA. During isometric contraction at a fixed ankle angle (~110°), pennation angle increased markedly from rest to MVC (≈15.5° to ≈33.6°), while fascicle length decreased by approximately 35%, with no significant change in the distance between aponeuroses. This architectural reorganization produced a 34.8% increase in PCSA from rest to MVC. Architectural changes were consistent across proximal, central, and distal regions, indicating homogeneous behaviour along the muscle belly. Ultrasound-derived architectural measurements in the cadaver showed strong agreement with direct anatomical measurements, validating ultrasound as an accurate non-invasive method for assessing muscle architecture. Overall, the study demonstrated that gastrocnemius medialis architecture is highly dependent on both joint angle and contraction intensity. The findings highlighted that cadaver-derived architectural data can only be extrapolated to in vivo conditions when muscle length is carefully matched, establishing a foundational methodological framework for subsequent in vivo ultrasound and biomechanical studies of human muscle function.
Simone Moroni et al. 2021 [33]	Anatomical basis of a safe mini-invasive technique for lengthening of the anterior gastrocnemius aponeurosis	10 fresh-frozen human cadaver specimens (10 donors: 8 males, 2 females; 5 left and 5 right lower limbs)	Multidisciplinary research team including podiatric surgeons with more than 6 years of experience in ultrasound-guided procedures and a clinical anatomist with over 10 years of experience in clinical anatomy and surgery	High-frequency ultrasound system with linear probe (17 MHz; SonoScape Medical Corp., Shenzhen, China Ultrasound-guided surgical instruments: 18-gauge needle and 50-cc syringe (for hydrodissection) V-shaped straight curettes (1–2 mm; 5 cm length) 3 mm hook knife (Acufex^®^) Buttoned probe Analog goniometer and caliper for ROM and anatomical measurements	All specimens were positioned in the supine decubitus position. Ankle joint range of motion (ROM) was measured with the knee fully extended using an analog goniometer, applying a standardized passive dorsiflexion force (≈2–5 kg). Prior to surgery, detailed sono-anatomical mapping was performed to identify the anterior gastrocnemius muscle aponeurosis, gastro-soleus interval, great saphenous vein, saphenous nerve, sural nerve branches, plantaris tendon, and myotendinous junctions. The ultrasound-guided gastrocnemius intramuscular aponeurosis release (GIAR) was performed through a single mini-portal (~2 mm), located proximal to the distal transection zone at the gastro-soleus medial interval. Hydrodissection was first carried out to create a safe working space between the anterior gastrocnemius aponeurosis and the soleus aponeurosis. Under continuous ultrasound guidance, progressively larger V-shaped curettes were introduced, followed by a retrograde hook knife to selectively transect the anterior gastrocnemius aponeurosis while preserving surrounding neurovascular structures. The plantaris tendon was identified and transected when present. Following completion of the procedure, all specimens underwent careful anatomical dissection to verify the completeness of aponeurosis release and to assess potential iatrogenic damage.	Primary measurements included: Ankle joint ROM (degrees) pre- and post-procedure Length and width of the aponeurotic gap after GIAR Thickness and medio-lateral width of the anterior gastrocnemius aponeurosis Portal length Qualitative assessment focused on the integrity of neurovascular structures (saphenous nerve, sural nerve branches, great saphenous vein).	Complete transection of the anterior gastrocnemius muscle aponeurosis was achieved in 10 out of 10 specimens, with a mean portal length of 2 ± 1 mm. Post-procedure anatomical dissection confirmed no injury to the saphenous nerve, sural nerve branches, great saphenous vein, or surrounding structures. The mean increase in ankle joint ROM after GIAR was 7.9 ± 1.1°, demonstrating effective lengthening of the gastrocnemius unit. The mean aponeurotic gap created by the release measured 12 ± 5 mm, while the mean dorso-ventral thickness of the anterior gastrocnemius aponeurosis was 1.3 ± 0.3 mm and its medio-lateral width 109 ± 11 mm. Overall, the study demonstrated that the ultrasound-guided GIAR technique allows precise, reproducible, and ultra-minimally invasive lengthening of the anterior gastrocnemius aponeurosis with a high safety profile. These findings provide a strong anatomical and methodological foundation for the clinical application of ultrasound-guided gastrocnemius lengthening procedures.
Stefano Bianchi et al. 2002 [16]	Central aponeurosis tears of the rectus femoris: sonographic findings	Cadaveric anatomical study: Rectus femoris muscles dissected from embalmed cadavers (bilateral thighs from one cadaver for dissection; axial sections from a second cadaver for US–anatomy correlation) In vivo healthy subjects: 20 healthy volunteers (14 males, 6 females; mean age: 31 years) Bilateral examination (40 thighs) Patient group (retrospective): 17 patients with acute anterior thigh injuries [16 males, 1 female; mean age: 26 years] Follow-up ultrasound available in 5 patients MRI correlation available in 8 patients	Experienced musculoskeletal radiologists from the University Hospital of Geneva and the University of Genoa, with expertise in musculoskeletal ultrasound and MRI, working in consensus for image interpretation	High-resolution B-mode ultrasound systems: HDI 5000 and HDI 3000 (ATL, Bothell, WA, USA) AU-4 Idea (Esaote, Genoa, Italy) Linear-array transducers (5–12 MHz and 10–13 MHz) Magnetic resonance imaging system: 2.0-T MRI scanner (Prestige, Elscint, Haifa, Israel) with pelvic coil	The study was conducted in three complementary phases. In the cadaveric phase, rectus femoris muscles were dissected to define the internal architecture and confirm the presence, orientation, and location of the central aponeurosis. Axial anatomical sections were obtained at proximal, middle, and distal thirds of the muscle and directly compared with corresponding in vitro ultrasound images to validate sonographic anatomy. In the in vivo healthy-subject phase, axial and sagittal ultrasound examinations were performed at the middle third of the rectus femoris with subjects lying supine, knee extended, and quadriceps relaxed. Additional images were obtained during isometric quadriceps contraction (hip flexed approximately 20°) to evaluate dynamic architectural changes. In the retrospective patient phase, ultrasound examinations of patients presenting with acute anterior thigh pain after sprinting or kicking were reviewed. Axial and sagittal scans were obtained with patients supine and the knee slightly flexed. Dynamic ultrasound during moderate muscle contraction was performed, and the contralateral side was examined for comparison. MRI was performed within 10 days of injury when available and used as the reference standard for lesion characterization.	Primary ultrasound assessments included: Visualization and morphology of the central aponeurosis Echogenicity and thickness of the aponeurosis Presence, size, and echotexture of peri-aponeurotic abnormalities Lesions were classified sonographically into three groups based on appearance and extent. MRI findings were used to confirm lesion type and extent when available.	Cadaveric dissection confirmed the central aponeurosis as a sagittally oriented fibrous band located within the proximal two-thirds of the rectus femoris muscle belly. In vitro and in vivo ultrasound consistently depicted the aponeurosis as a curvilinear hyperechoic structure, best visualized in the axial plane. The mean aponeurosis thickness in healthy subjects was approximately 1.5 mm. Ultrasound identified the central aponeurosis in 100% of healthy thighs, with sonographic anatomy closely matching cadaveric and MRI findings. In the injured patient group, three sonographic patterns were identified: Nine small partial tears (irregular hyperechoic areas surrounding an intact central aponeurosis, consistent with hemorrhagic infiltration; Larger partial tears (n = 7): mixed hypoechoic–hyperechoic regions with muscle swelling and globular enlargement, consistent with hematoma and tissue infiltration; Complete myotendinous disruption (n = 1): fluid-filled gap between retracted muscle ends with preservation of aponeurotic continuity. MRI findings, available in eight patients, confirmed the ultrasound-based classification in all cases. Follow-up ultrasound demonstrated hyperechoic, poorly defined regions consistent with fibrotic scar formation around the central aponeurosis. Overall, the study demonstrated that sonography can reliably depict both the normal internal architecture of the rectus femoris and pathological changes involving the central aponeurosis. The strong correlation with MRI findings, combined with low cost and wide availability, supports ultrasound as the first-line imaging modality for diagnosis and follow-up of rectus femoris central aponeurosis injuries.
Hiroshi Akima et al. 2022 [20]	Effect of subcutaneous adipose tissue and muscle thicknesses on rectus femoris and vastus intermedius ultrasound echo intensities: a cadaver study	11 legs from 7 human cadavers fixed using Thiel’s method (4 ♂, 3 ♀ in supine position	Ultrasound measurements performed by a single investigator (KY), highly trained and experienced in musculoskeletal ultrasound imaging.	A real-time B-mode ultrasonography system (LOGIQ e; GE Healthcare, Chicago, IL, USA) with an 8 to 12 MHz linear array probe (width, 3.8 cm). Ultrasound acquisition parameters: Frequency: 10 MHz Gain: 70 dB Imaging depth: 2 or 3 cm Number of focal points: 1 (positioned at the top of the image)	Transverse ultrasound images of the quadriceps femoris were obtained at the anterior mid-thigh of cadavers positioned supine, with the hip and knee joints placed in standardized anatomical alignment. Ultrasound imaging was performed under three sequential experimental models designed to isolate the effects of superficial and deep tissues on echo intensity (EI): Model 1: intact tissues, replicating standard in vivo ultrasound measurements in living humans; Model 2: measurement after complete removal of subcutaneous adipose tissue (SCAT) from the anterior thigh; Model 3: measurement after removal of both SCAT and the rectus femoris (RF), leaving the vastus intermedius (VI) fully exposed. All dissections were performed carefully to avoid deformation of the remaining tissues. Ultrasound images were subsequently analyzed offline to quantify SCAT thickness, RF and VI muscle thickness, and echo intensity values.	Echo intensity (EI; arbitrary units, a.u.) of the rectus femoris and vastus intermedius was quantified using grayscale analysis of B-mode images. Subcutaneous adipose tissue thickness and muscle thicknesses (RF and VI) were measured directly from ultrasound images using image analysis software v1.53 (National Institutes of Health, Bethesda, MD, USA).	In the intact condition (Model 1), SCAT thickness measured 0.36 ± 0.19 cm. Rectus femoris thickness was 0.72 ± 0.42 cm in Model 1 and remained unchanged after SCAT removal in Model 2 (0.69 ± 0.35 cm), indicating preserved RF morphology following superficial tissue removal. Vastus intermedius thickness was consistent across experimental conditions (0.45 ± 0.20 cm in Model 1; 0.45 ± 0.21 cm in Model 2; 0.43 ± 0.22 cm in Model 3), confirming that progressive tissue removal did not alter deeper muscle structure. Rectus femoris echo intensity increased significantly after removal of SCAT, rising from 69.4 ± 20.2 a.u. in Model 1 to 83.7 ± 15.8 a.u. in Model 2, demonstrating marked attenuation of ultrasound signal by superficial adipose tissue. Vastus intermedius echo intensity exhibited a progressive increase with stepwise removal of overlying tissues: EI was not reported for Model 1, increased to 86.1 ± 23.3 a.u. in Model 2, and reached 102.2 ± 21.8 a.u. in Model 3, highlighting the strong dependence of deep muscle EI on the thickness and composition of overlying structures. Multiple regression analyses revealed that SCAT thickness (*p* = 0.036) and RF thickness (*p* = 0.001) were significant predictors of RF EI, whereas VI EI was primarily determined by RF thickness (*p* = 0.035), with no independent contribution from SCAT or VI thickness. Overall, the study demonstrated that removal of superficial anatomical layers leads to substantial and systematic increases in muscle echo intensity. These findings indicate that ultrasound EI cannot be interpreted independently of tissue depth and overlying anatomy, providing essential methodological and interpretative guidance for assessing muscle quality in both research and clinical ultrasound imaging.
Alexandre Moraux et al. 2015 [21]	An anatomical study of the indirect tendon of the rectus femoris using ultrasonography	4 hips from 2 embalmed cadavers (1 male, 1 female; mean age: 67 years) 1 additional formaldehyde-embalmed cadaver hip sectioned for histology 4 additional cadaver hips from 2 formaldehyde-embalmed cadavers (mean age: 70 years) for ultrasound–dissection correlation. In vivo ultrasound study: 20 healthy adult volunteers (13 males, 7 females; age range 19–60 years; mean age 34.1 years) Bilateral examination (40 hips)	Two experienced musculoskeletal radiologists (8 and 2 years of experience), working in consensus for both cadaveric and in vivo ultrasound assessments, with histological validation performed by a senior pathologist (25 years of experience)	High-resolution ultrasound systems: LOGIQ E9 (General Electric, UK) Linear-array transducers (6–15 MHz) Ultrasound-guided spinal needles (21G) for cadaveric validation Microtome and histological equipment for tendon sectioning and analysis Goniometer for probe angulation measurement	The study was conducted in three sequential phases. In the cadaveric anatomical phase, proximal rectus femoris insertions were carefully dissected to define the morphology, course, and anatomical relationships of the indirect tendon. Additional specimens were sectioned into axial oblique slices aligned with the tendon’s long axis, followed by histological analysis to characterize tendon microstructure. In the cadaveric ultrasonographic validation phase, the indirect tendon was identified using ultrasound via a novel lateral approach. Under real-time ultrasound guidance, spinal needles were advanced through the direct tendon into the indirect tendon. Subsequent anatomical dissection along the needle trajectory was performed to verify accurate sonographic localization. In the in vivo ultrasound phase, healthy volunteers were examined in the supine position with the hip in neutral alignment. The indirect tendon was assessed bilaterally using a lateral axial oblique ultrasound approach, with the probe positioned approximately 30° relative to the axial plane, just lateral to the anterior inferior iliac spine (AIIS). Long- and short-axis views were obtained to evaluate tendon visibility, echogenicity, and morphology.	Primary ultrasound measurements included: Visibility of the indirect tendon Tendon length (mm) Tendon width (mm) Tendon thickness (mm) Probe angulation relative to the axial plane (degrees) Secondary qualitative assessments included tendon echogenicity and identification of the hyperechoic fatty space separating the tendon from the lateral iliofemoral ligament.	Anatomical dissection and histological analysis confirmed that the indirect tendon is a thin, flat collagenous structure arising from the posterosuperior acetabular ridge and posterior capsule, extending distally to merge with the direct tendon to form the conjoined tendon. Histology demonstrated dense regular connective tissue with parallel collagen fiber organization. Ultrasound–guided needle placement in cadavers confirmed accurate sonographic depiction of the indirect tendon, with needles located within or immediately adjacent to the tendon in all specimens. In vivo, the indirect tendon was successfully identified in 100% of hips using the lateral ultrasound approach. The tendon appeared as a convex, fibrillar structure beneath the gluteus minimus muscle and was consistently separated from the lateral iliofemoral ligament by a narrow hyperechoic fatty space. Mean indirect tendon dimensions were 33.6 mm in length, 9.88 mm in width, and 4.35 mm in thickness, with a mean probe angulation of 29.7° relative to the axial plane. No significant differences were observed between sides or sexes. Overall, the study demonstrated that the indirect tendon of the rectus femoris—previously considered poorly accessible to ultrasound—can be reliably and reproducibly visualized using a lateral approach. These findings establish a strong anatomical and methodological basis for the clinical ultrasound assessment of proximal rectus femoris injuries, potentially reducing reliance on MRI and enabling earlier diagnosis in sports and musculoskeletal medicine.
Taiki Kodesho et al. 2021 [18]	Relationship between shear elastic modulus and passive force of the human rectus femoris at multiple sites: A Thiel soft-embalmed cadaver study	4 rectus femoris (RF) muscles harvested from Thiel soft-embalmed human cadavers (mean age at death: 81 years; range: 60–98 years)	Research team from Sapporo Medical University and Gunma University with expertise in musculoskeletal biomechanics, anatomy, and ultrasound shear-wave elastography	Ultrasound shear-wave elastography system (Aixplorer Ver. 12; Supersonic Imagine, Aix-en-Provence, France) Linear-array ultrasound transducer (4–15 MHz, SL15-4) Custom-built passive loading device with clamps, pulley, and cable system (Uchida Systems Co., Ltd., Tokyo, Japan) Ultrasound diagnostic echo pad, 30 mm thickness (Echo PAD; Yasojima Proceed Co., Ltd., Japan) Flexible arm to fix and stabilize probe position	Four rectus femoris muscles were carefully dissected from Thiel soft-embalmed cadavers, preserving both the proximal tendon (anterior inferior iliac spine and acetabulum) and the distal tendon (upper margin of the patella). Each specimen was mounted on a custom-built loading apparatus that applied standardized passive tensile loads to the distal tendon via a pulley–weight system, while the proximal tendon was rigidly fixed. Passive loads were increased stepwise from 0 to 600 g in 60 g increments under controlled environmental conditions (room temperature ≈22 °C; humidity ≈40%). An ultrasound echo pad was placed beneath the muscle to ensure consistent acoustic coupling and minimize friction during elongation. The ultrasound transducer was aligned longitudinally with the muscle fibers and secured using a flexible arm to maintain a constant orientation and measurement location throughout the experiment. Shear-wave elastography (SWE) measurements were obtained at three predefined regions along the muscle length: proximal (25%), central (50%), and distal (75%) positions between the proximal origin and distal tendon. For each load level, elastographic images were acquired approximately 5 s after load application to allow stabilization, and measurements were repeated twice. A 1 min rest interval was imposed between successive load applications to minimize creep and hysteresis effects.	Muscle shear elastic modulus (kPa) was quantified from SWE images using a rectangular region of interest (20 mm × 5 mm). Young’s modulus values provided by the SWE system were converted to shear modulus by dividing by three to account for muscle anisotropy assumptions. Force–elasticity relationships were derived by linear regression between applied passive load and shear modulus at each muscle region. Test–retest reliability was assessed using intraclass correlation coefficients (ICC), coefficient of variation (CV), and standard error of measurement (SEM).	At all three measurement sites, shear modulus measured in the longitudinal direction exhibited a strong, near-linear relationship with applied passive force. Mean coefficients of determination (R^2^) were 0.927 ± 0.081 at the proximal region, 0.978 ± 0.018 at the central region, and 0.942 ± 0.010 at the distal region (all *p* < 0.01). Shear modulus measurements obtained in the transverse plane did not consistently correlate with passive force, highlighting the anisotropic mechanical behaviour of skeletal muscle and the importance of probe alignment with muscle fibers. Test–retest reliability of longitudinal SWE measurements was excellent across all regions (mean ICC ≥ 0.987), with low coefficients of variation (<2%) and small standard errors of measurement (<0.6 kPa), confirming high measurement reproducibility. Overall, the study demonstrated that shear-wave elastography performed along the longitudinal axis of the rectus femoris can reliably track changes in passive muscle force at proximal, central, and distal sites. These findings provide strong experimental evidence supporting SWE as a valid indirect method for estimating region-specific passive muscle forces in human skeletal muscle and contribute to improved biomechanical understanding of intramuscular heterogeneity relevant to injury risk, rehabilitation, and musculoskeletal modeling.
Gonzalo J. Delgado et al. 2002 [22]	Tennis leg: Clinical US study of 141 patients and anatomic investigation of four cadavers with MR imaging and US	Cadaveric anatomical study: 4 non-embalmed fresh-frozen human cadaver legs (4 males; age range at death: 78–86 years; mean age: 80.2 years) Clinical ultrasound study: 141 patients referred with a clinical diagnosis of tennis leg (109 males, 32 females; age range: 22–82 years; mean age: 45 years)	Experienced musculoskeletal radiologists from the University of California, San Diego (UCSD) and Veterans Affairs Medical Center, with expertise in musculoskeletal ultrasound, MRI, and lower-limb anatomy, working in consensus for image interpretation	Ultrasound systems: HDI 5000 and HDI 3000 (Advanced Technology Laboratories, Bothell, WA, USA) Linear-array broadband transducers (4–7.5 MHz and 5–12 MHz) Magnetic resonance imaging system: 1.5-T superconducting MRI scanner (Signa; GE Medical Systems, Milwaukee, WI, USA) Dedicated knee coil	The study consisted of a combined cadaveric anatomical investigation and a retrospective clinical ultrasound analysis. In the cadaveric phase, four fresh-frozen human legs were thawed and examined using both ultrasound and MRI to delineate the anatomy of the posterosuperficial compartment of the calf, with particular emphasis on the plantaris muscle and tendon and their relationship to the gastrocnemius and soleus muscles. Ultrasound examinations were performed in longitudinal and transverse planes, followed by T1-weighted MRI acquisitions in axial, sagittal, and coronal planes. After imaging, specimens were refrozen and either sectioned in planes corresponding to MRI images or dissected to allow direct gross anatomical correlation. In the clinical phase, ultrasound images from 141 patients presenting with acute calf pain consistent with tennis leg were retrospectively reviewed. Examinations were performed with patients in the prone position, using longitudinal and transverse scanning planes. Images were analyzed for integrity of the medial gastrocnemius, soleus, and plantaris musculotendinous units, presence of fluid collections between aponeuroses, and evidence of deep venous thrombosis. The contralateral limb was examined for comparison.	Primary ultrasound assessments included: Presence and location of musculotendinous ruptures Integrity of the medial head of the gastrocnemius, plantaris tendon, and soleus muscle Presence and distribution of fluid collections between aponeuroses Sonographic signs of deep venous thrombosis Findings were categorized based on lesion type and anatomical location	Cadaveric imaging and dissection demonstrated that ultrasound and MRI could reliably distinguish the plantaris musculotendinous unit from surrounding structures and accurately depict its anatomical course relative to the medial gastrocnemius and soleus muscles. In the clinical cohort, ultrasound findings revealed that partial rupture of the medial head of the gastrocnemius muscle at the musculotendinous junction was the most frequent abnormality, observed in 94 of 141 patients (66.7%). Fluid collection between the aponeuroses of the medial gastrocnemius and soleus muscles without demonstrable muscle rupture was identified in 30 patients (21.3%). Isolated rupture of the plantaris tendon was rare, occurring in only 2 patients (1.4%), while partial rupture of the soleus muscle was observed in 1 patient (0.7%). Deep venous thrombosis was identified in 14 patients (9.9%) as an isolated finding and in an additional 7 patients (5.0%) in association with other sonographic abnormalities, underscoring the importance of systematic venous evaluation in patients with suspected tennis leg. Overall, the study demonstrated that abnormalities of the medial gastrocnemius muscle are far more common than injuries to the plantaris tendon in patients with clinical tennis leg. Ultrasound proved to be an effective first-line imaging modality for diagnosis, anatomical differentiation, and exclusion of serious mimics such as deep venous thrombosis, providing a strong anatomical and clinical basis for the modern understanding of tennis leg pathology.
D. Tosovic et al. 2016 [19]	Anatomy of the long head of biceps femoris: An ultrasound study	In vivo ultrasound study: 19 healthy young male participants (mean age: 21.6 ± 2.3 years; age range: 18–30 years) Cadaver validation study: 6 lower limbs from 3 embalmed male cadavers (mean age at death: 76 years; range: 65–86 years)	Research team from the University of Queensland (Australia) and the University of Otago (New Zealand), with expertise in anatomy, musculoskeletal ultrasonography, and muscle architecture	Real-time B-mode ultrasound system (Sonoline Antares™; Siemens Medical Solutions, Malvern, PA, USA) Linear-array ultrasound probe (VF10–5; 5–10 MHz) with extended field-of-view imaging capability Flexible tape measure for surface anatomical measurements Digital calipers for cadaveric dissection measurements Standard protractor for pennation angle assessment during dissection	In the in vivo study, participants were positioned prone with the lower limbs extended and in neutral rotation. Ultrasound scans were performed bilaterally along the long head of the biceps femoris (BFlh). The proximal origin (ischial tuberosity), distal insertion (head of the fibula), and the most proximal and distal extents of muscle fiber insertion were identified and marked on the skin. Using these landmarks, total muscle–tendon length, muscle length, proximal musculotendinous junction (MTJ) length, distal MTJ length, and distal free tendon length were measured. Ultrasound images were acquired at four standardized locations along the muscle length—30%, 50%, 70%, and 90% of total muscle length—to assess segmental muscle architecture. At each site, muscle thickness was measured from transverse images, while fascicle length and pennation angle were measured from longitudinal images. Fascicle length was defined as the distance between superficial and deep aponeuroses, and pennation angle as the angle between the superficial aponeurosis and a visible fascicle. In the cadaver validation study, the same ultrasound protocol was applied. Pins were inserted at each measurement site to mark locations for subsequent dissection. Following dissection, architectural parameters were measured directly using calipers and a protractor, allowing comparison between ultrasound-derived and anatomical measurements.	Primary architectural parameters included: – Muscle thickness (mm) Fascicle length (mm) Pennation angle (degrees) Additional measurements included total muscle–tendon length, muscle length, proximal and distal MTJ lengths, distal free tendon length, and fascicle length–to–muscle length ratio (FL/ML). Intra-rater reliability was assessed for repeat scans, repeat image analyses, and repeat dissection measurements using intraclass correlation coefficients (ICC).	Ultrasound revealed that the long head of the biceps femoris exhibits non-uniform architecture along its length. The distal region (90% of muscle length) displayed significantly shorter fascicles and larger pennation angles compared with proximal regions, whereas the proximal region (30% of muscle length) exhibited longer fascicles and smaller pennation angles, indicating greater excursion potential. The middle regions (50% and 70% of muscle length) were significantly thicker than both proximal and distal regions, consistent with a fusiform muscle shape and suggesting a greater contribution to force generation. Intra-rater reliability for ultrasound-derived architectural measurements was good to excellent (ICC generally > 0.80), and reanalysis of ultrasound images showed excellent repeatability for fascicle length and pennation angle (ICC ≈ 0.97). Comparison between ultrasound and cadaveric dissection demonstrated excellent agreement for most architectural parameters, validating ultrasound as an accurate method for assessing BFlh architecture. Pennation angle and MTJ length showed lower agreement, likely due to the complex three-dimensional geometry of these structures. Overall, the study demonstrated that the long head of the biceps femoris is architecturally heterogeneous, with proximal segments better suited for muscle excursion and distal segments optimized for force production. These segmental differences provide a plausible anatomical explanation for the high incidence of strain injuries at the proximal musculotendinous junction and establish a detailed reference for interpreting ultrasound images in both clinical and sports medicine contexts.
Eleftherios Kellis et al. 2009 [17]	Validity of architectural properties of the hamstring muscles: Correlation of ultrasound findings with cadaveric dissection	6 lower limbs from 3 embalmed male human cadavers (mean age at death: 68.3 years; range: 65.4–71.1 years)	Multidisciplinary research team from the Aristotle University of Thessaloniki (Departments of Physical Education and Sport Sciences, Orthopaedics, and Anatomy), with expertise in muscle architecture, biomechanics, and musculoskeletal ultrasonography	Real-time B-mode ultrasound system (SSD-3500; ALOKA, Japan) Linear-array ultrasound probe (10 MHz) Transmission gel for acoustic coupling Digital video-based motion analysis software (Max Traq Lite v2.09; Innovision Systems, Columbiaville, MI, USA) for image digitization High-definition digital camera for cadaveric architectural measurements Precision calipers and tape measure for direct anatomical measurements	The long head of the biceps femoris (BFlh) and the semitendinosus (ST) muscles were examined bilaterally in embalmed cadavers positioned in the anatomical position, with the hip and knee joints fixed at 0° (full extension). Ultrasound scanning was performed along the longitudinal axis of each muscle. The proximal origin at the ischial tuberosity and distal insertions (fibular head for BFlh; gracilis tendon/fascia cruris for ST) were first identified using axial and longitudinal scans and marked on the skin. Continuous ultrasound sweeps were then performed from distal to proximal to visualize the entire muscle length and identify the distal myotendinous junction (MTJ). Standardized ultrasound images were acquired at approximately 10%, 30%, 50%, and 70% of the curved path between the distal MTJ and proximal origin. From mid-belly images, muscle thickness, pennation angle, and fascicle length were measured. When full fascicles were not visible, fascicle length was estimated using trigonometric equations based on pennation angle and muscle thickness. Following ultrasound assessment, cadaveric dissection was performed. The hamstring muscles were carefully removed from their attachments, longitudinally incised along the fiber direction, and architectural parameters were measured directly at locations corresponding to ultrasound measurement sites. Fascicles were teased apart to determine true fascicle length, and pennation angle was measured from high-resolution photographs.	Primary architectural parameters included: Fascicle length (FL, cm) Pennation angle (PA, degrees) Muscle thickness (MT, cm) Additional measurements included: Muscle length (ML, cm) Distal tendon length (DTL, cm) Validity of ultrasound measurements was assessed by comparison with direct dissection using intraclass correlation coefficients (ICC), standard error of measurement (SEM), and smallest detectable difference (SDD).	Ultrasound-derived measurements demonstrated a high level of agreement with direct anatomical measurements for both muscles. For the long head of the biceps femoris, ICC values ranged from 0.905 to 0.992, while for the semitendinosus ICC values ranged from 0.774 to 0.972, indicating good to excellent validity across architectural parameters. Mean ultrasound measurement errors relative to dissection were small: Muscle thickness: 0.09–0.14 cm Pennation angle: 1.01–1.31° Fascicle length: 0.92–1.71 cm No significant differences were observed between ultrasound and dissection for most variables. A small but significant underestimation of semitendinosus fascicle length by ultrasound was detected, attributed to the indirect estimation of fascicle length when full fascicles were not visible. Muscle length and distal tendon length measurements obtained by ultrasound showed high agreement with dissection (ICC > 0.90), although distal biceps femoris tendon length was slightly underestimated by ultrasound. Overall, the study demonstrated that B-mode ultrasound provides a valid and reliable alternative to cadaveric dissection for assessing key architectural properties of the hamstring muscles. These findings support the use of ultrasound for in vivo evaluation of hamstring muscle morphology, with important implications for biomechanics, injury assessment, rehabilitation, and musculoskeletal modeling.
